# Structurally Colored Thin Films Based on Acetylated Lignin Nanoparticles

**DOI:** 10.1021/acsnano.4c16679

**Published:** 2025-07-01

**Authors:** Ravi Shanker, Anran Mao, Longzhu Liu, Aseem Salhotra, Yuxiao Cui, Bang An, Magnus P. Jonsson, Anna J. Svagan

**Affiliations:** 1 Dept. of Fibre and Polymer Technology, 7655 Royal Institute of Technology (KTH), Stockholm SE-100 44, Sweden; 2 Laboratory of Organic Electronics, Department of Science and Technology, Linköping University, Norrköping SE-601 74, Sweden

**Keywords:** structural colors, lignin, nanoparticles, thin-film interference, membrane emulsifications, sorption

## Abstract

In nature, colors can originate from pigments or structural effects, with the latter producing brilliant hues through the interference of light with nanoscale structures. This study describes a feasible strategy to achieve structurally colored films based on acetylated lignin nanoparticles. Lignin nanoparticles were prepared by using membrane emulsification and subsequently self-assembled into multilayered films on silicon substrates through an evaporative process. These films exhibit vivid structural colors resulting from thin-film interference, with hues that vary with film thickness. Spectroscopic reflectance measurements and structural analysis reveal a wide range of colors spanning across the visible spectrum. The observed colors are ascribed to interference effects and could be modeled using the transfer matrix method. Furthermore, we demonstrate that increasing relative humidity causes clear color shifts associated with reflectance peak position changes.

## Graphical abstract

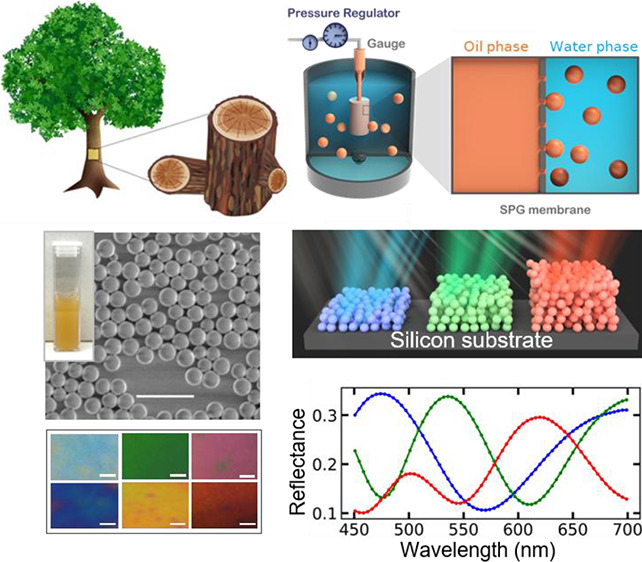

## Introduction

Structural coloration (SC) results from the interaction of light from micro- and nanoscale materials, creating colors via effects like scattering, Bragg diffraction, and thin-film interference.^1–5^ Unlike dyes and pigments that rely on material absorption features, SC depends on material structure to produce vivid hues, through reflection, transmission, absorption, and scattering.^6,7^ Structural colors are often more stable than dyes and pigments, typically resisting fading, while avoiding potential environmental and health risks associated with some dyes and pigments.^8,9^ SC found in organisms ranging from bacteria,^10^ plants,^11^ insects, and crustaceans^12^ to bird plumage can display a spectrum of colors. Researchers, inspired by these natural examples, have replicated SC using a synthetic colloidal^13–17^ system such as polystyrene (PS), PMMA, SiO_2_, and TiO_2_ and in natural biomolecules like melanin, cellulose, chitin, and xanthommatin.^12,18–23^ Plant biomass polymers, like cellulose, hemicellulose, and lignin hold huge potential for conversion into high-value products, including SC. With the rise of sustainable materials, the efficient use of these biomolecules, especially cellulose nanocrystals (CNCs) and hydroxypropyl cellulose (HPC), has gained significant attention in SC.^24,25^ On the other hand, lignin, a major component of plant biomass,^26^ remains largely unexplored for optical applications. While primarily considered a byproduct of the pulp and paper industry^27^ and often burnt for energy, it is a renewable and cost-effective raw material. Lignin can be extracted from plant-based sources, including wood and agricultural side-streams, using green extraction pathways,^28^ and its intrinsic aromatic nature, biodegradability, nontoxicity, and biocompatibility make it promising for advanced applications beyond energy generation such as SC-based humidity-responsive sensors, decorative and anticounterfeiting materials, and smart packaging. Although lignin nanoparticles (LNPs) have previously been reported in literature, the focus predominantly remains on its role as functional additive in composites and adhesives, where the monodispersity of the LNPs is less critical.^29–32^ A few approaches have been explored to create lignin-based structural colors, each with distinct advantages and limitations. Centrifugation-assisted assembly has been used to produce photonic structures from LNPs, allowing size-based classification for color variation. However, achieving a uniform single color remains challenging due to variations in particle packing and a high polydispersity index (0.19), while yield efficiency remains low.^33,34^ Similarly, cascade and density gradient centrifugation enable narrow size distributions but require multiple steps. Photonic colors using this method were primarily demonstrated in the wet state, with color stability after drying remaining unclear.^35,36^ Solvent fractionation and controlled self-assembly have been used to generate biophotonic coatings, but precise nanoparticle size control is needed for color tuning, limiting scalability. Additionally, acetylated LNPs with polydispersity indices (PDIs) above 0.1 have successfully been used for transparent coatings with superhydrophilic antifogging properties, which relied on a layer-by-layer deposition using poly-l-lysin, to precisely control thickness. However, the introduction of poly-l-lysin makes the underlying color formation mechanism dependent on the electrostatic assembly of the poly-l-lysin and LNPs.^37^ In contrast, our membrane emulsification method achieves a narrow size range of nanoparticles while directly forming thin films without the need for binders. The ability to tune the nanoparticle size and film thickness is key when controlling the structural color formation.

Building on previous studies on self-assembled LNPs, we introduce an alternative approach. Using membrane emulsification, we produce LNPs that self-assemble into thin films, creating structural colors through thin-film interference.

We used acetylated lignin, which reduces brown tones, by modifying chromophores while preserving UV absorption, making it suitable for vibrant SC.^38^ Acetylation is a common modification in plant cell walls and plays a key role in tailoring physicochemical properties.^39^ Here, acetylation enables the utilization of the membrane emulsification technique for the preparation of monodispersed LNPs, which were then assembled into structurally colored films. The monodispersed LNPs are prepared from acetylated Kraft lignin.^40,41^ These acetylated LNPs are produced by passing fractionated, chemically modified lignin through a membrane to form oil droplets of controlled size and eventually solidify into LNP colloids in water. Subsequently, we use evaporative self-assembly to pack LNPs into thin films, forming random, closely packed layers, typically consisting of just a few layers (1–5) that produce vivid structural colors; see Scheme 1. Applying a thin-film interference model could explain the origins of these colors. Comprehensive analysis using microreflectance spectroscopy, optical microscopy, and electron microscopy is performed. Taking advantage of the moderate swelling capability of these LNPs in the presence of water vapor, we demonstrate their sensitivity to humidity. This work highlights the potential of LNPs in the production of sustainable structural colors.

**Scheme 1:**
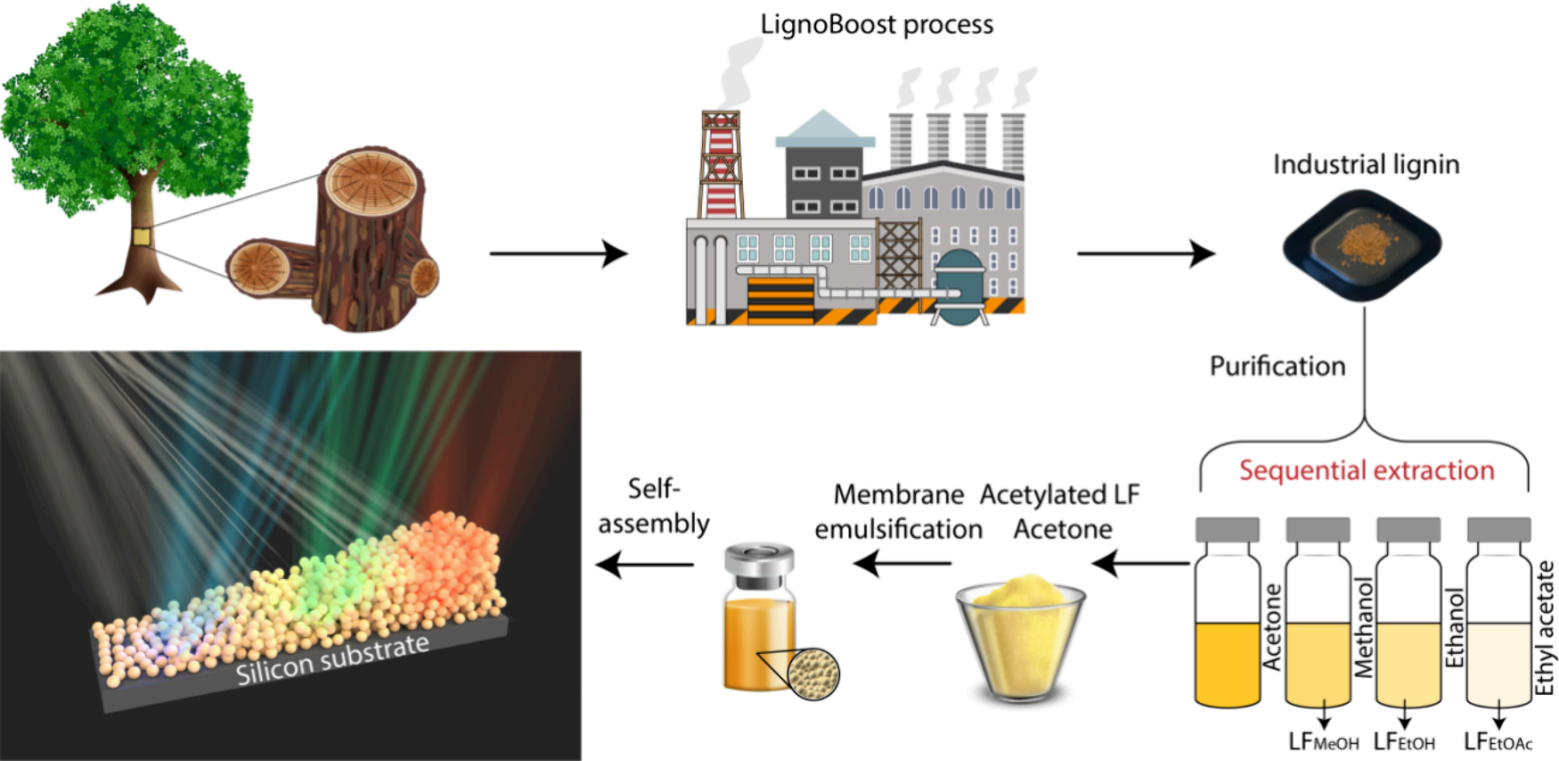
Fabrication-Steps of Structurally Colored Thin Films Based on Acetylated LNPs^a^

a Industrial softwood lignin is obtained from the LignoBoost process, and the lignin is sequentially fractionated using the following organic solvents: ethyl acetate (E_tOAc_), ethanol (EtOH), methanol (MeOH), and acetone, yielding fractionated lignin (LF) extracts (LF_EtOAc_, LF_EtOH_, LF_MeOH_, and LF_Acetone_, respectively). Only the acetone-soluble lignin fraction (LF_Acetone_) is used in the present study. The lignin is acetylated to enhance its solubility in an organic solvent and then converted into nanoparticles. Using evaporation-induced self-assembly, the acetylated LNPs form thin, structurally colored lignin colloidal films.

## Results and Discussion

Lignin’s aromatic nature, biodegradability, nontoxicity, and biocompatibility make it a valuable material. However, its molecular heterogeneity poses challenges, as the unpurified lignin contains molecules with varying solubilities,^42^ which leads to differences in physicochemical properties at the water/oil interface.^43^ This presents challenges for established methodologies to produce monodispersed LNPs for structural color applications. Often, these studies^44^ have chosen to circumvent the substantial challenges associated with the production of monodispersed LNPs. In turn, monodispersed nanoparticles are often considered important in SC systems, particularly for those relying on the particle arrangement. The lignin’s heterogeneous molecular structure, which includes a variety of molecular weights and functional groups, requires solvent fractionation to isolate specific fractions that can be consistently dissolved and processed.^42^ This fractionation of lignin is essential prior to generating uniform and monodispersed particles.^43^ Here, we used acetylation to improve the solubility of lignin in organic solvents, which is essential prior to the membrane emulsification step. By conversion of hydroxyl (−OH) groups into acetoxy (−OCOCH_3_) groups, acetylation increases the hydrophobicity of lignin, enhancing its dispersion in the organic oil phase. Using the membrane emulsification technique, an oil-in-water emulsion with well-defined oil-droplet sizes is formed, which in turn contributed to the formation of uniform nanoparticles.^45–47^ This ensures more consistent film quality and reproducible optical properties, making acetylated lignin a valuable material for photonic applications.^48^ The membrane emulsification method has shown great promise in creating micrometer-sized lignin particles with high precision.^43,49,50^ The membrane emulsification method utilizes a ceramic membrane with pores of known sizes. By extruding an acetylated lignin solution in chloroform, a liquid phase (the dispersed phase) is passed through a hydrophilic membrane under controlled pressure, into another immiscible liquid phase (the continuous phase) consisting of sodium dodecyl sulfate surfactant (SDS) and water. This forms droplets in an aqueous phase, which detach from the membrane and disperse into the continuous phase, forming an emulsion (Figure 1a and Figure S1). The type of emulsion created depends on the wetting property of the membrane. For instance, we can prepare oil-in-water (O/W) emulsions using hydrophilic membranes, whereas water-in-oil (W/O) emulsions require hydrophobic membranes.^51–53^ After droplet formation, chloroform was allowed to evaporate overnight, forming solid lignin particles. Figure 1b shows the homogeneous dispersion of Kraft lignin dissolved in chloroform (vial (i)) and LNPs in milli-Q water (vial (ii), respectively.

**Figure 1:**
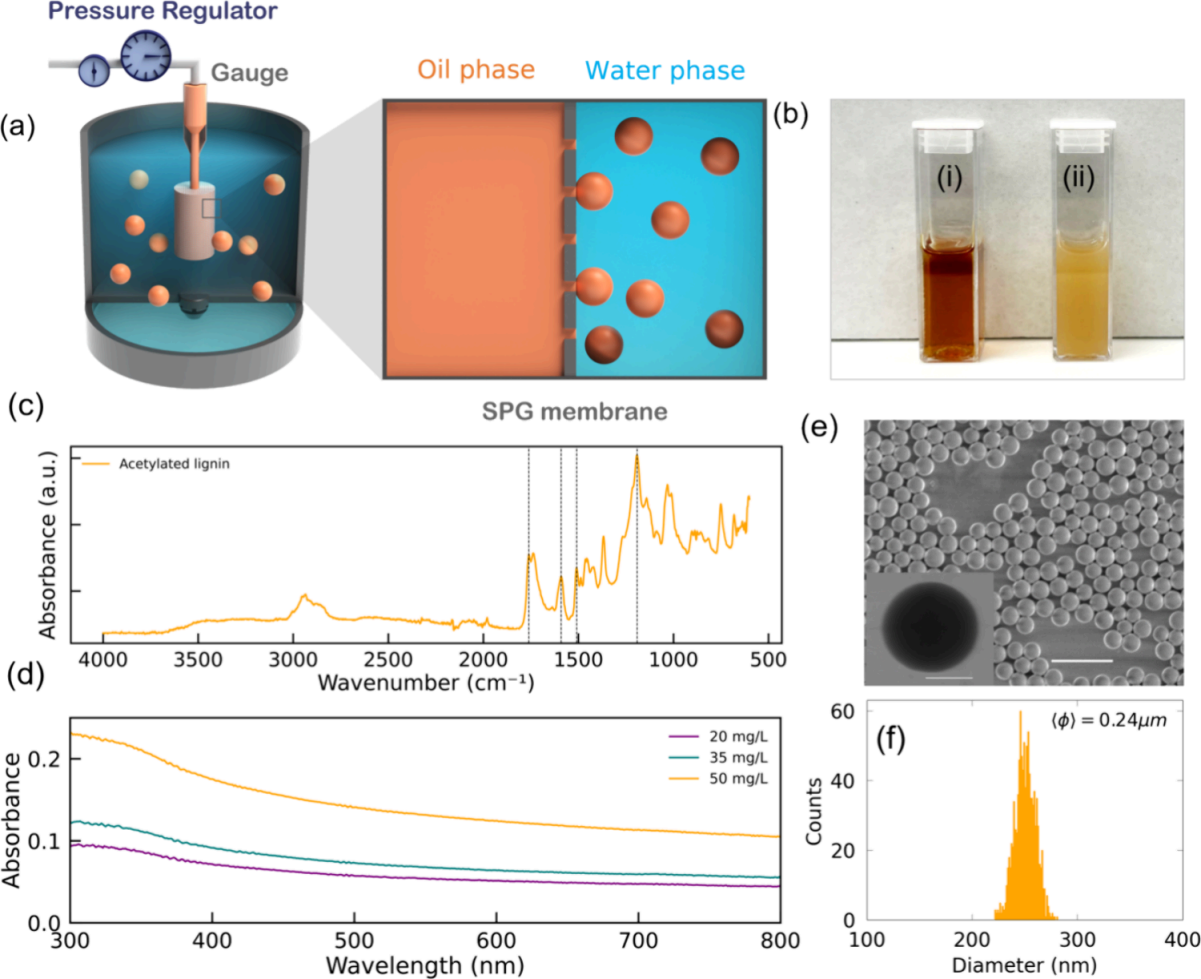
(a) Schematic diagram showing the key components involved in the membrane emulsification-based LNPs preparation. (b) Digital photo of homogeneous dispersion of Kraft lignin in the chloroform solvent (left) and right photo (dark brown color, vial (i)) shows the dispersed solution of LNPs (0.05 wt % in water, vial (ii)). (c) Fourier-transform infrared (FT-IR) spectra for acetylated lignin. (d) UV–vis absorption spectra of acetylated LNPs. (e) SEM micrograph of LNPs with a scale bar of 1 μm. The bottom-left inset shows transmission electron microscopy image of a colloidal LNP. Scale bar: 0.125 μm. (f) Histogram of the size distribution of LNPs measured by SEM, with an average particle diameter of 0.24 μm.

The FT-IR spectra for acetylated lignin, shown in Figure 1c (Figure S2), was consistent with our previous publications.^43,49,50^ Typical bands (highlighted by vertical dashed lines) include the one around 1761 cm^–1^, corresponding to the carbonyl (−C=O) stretching in acetoxy groups and the band at 1191 cm^–1^, due to the stretching of C–O in the acetylated phenolic hydroxyl groups,^54^ confirming successful acetylation. Additionally, aromatic skeletal vibrations are observed at 1591 and 1509 cm^–1^.^55^ The broadband around 3400 cm^–1^, associated with hydroxyl groups, shows reduced intensity, indicating that most of the remaining hydroxyl group have reacted (compared with the FT-IR spectra for the raw acetone-fraction of lignin, Figure S2 in SI). The UV–visible absorption spectrum of LNPs shows a strong UV absorbance that gradually decreases at longer wavelengths (Figure 1d). This behavior arises from chromophores, including phenolic hydroxyls, carbonyls, and conjugated double bonds, enabling lignin to effectively absorb ultraviolet radiation. Although acetylation modifies chromophore to reduce brown coloration, the remaining chromophores still contribute effectively to UV absorption, making it promising for UV-shielding applications.^56,57^ As shown in previous studies,^43^ this approach produced highly monodispersed particles that here have an average diameter of ϕ ∼0.24 μm, confirmed by scanning electron microscopy (SEM) micrographs (Figure 1e). This also aligns with transmission electron microscopy (TEM) micrograph data shown in the bottom-left inset of Figure 1e. Dynamic light scattering (DLS) measurements (Figure S3) further confirmed a monomodal size distribution with a low polydispersity (PDI = 0.07). A histogram from SEM imaging illustrates the particle size distribution (Figure 1f). The particle size is adjustable by using ceramic membranes with different pore sizes and the concentration of the dispersed phase, with smaller pore sizes and more dilute lignin concentrations in chloroform producing smaller particles.^58^ Additionally, due to the bulk nature of membrane emulsification, we could synthesize a large quantity of particles ∼10^12^ per batch. The surfactant (SDS) was removed by using dialysis.

Driven by interparticle interactions, colloidal self-assembly processes have progressed significantly.^59–64^ The colloidal crystal generally self-assembles with a face-centered-cubic (fcc) lattice, often with the close-packed [111] plane parallel to the substrate, although local variations occur depending on specific experimental conditions.^65–67^ We prepared colored films of LNPs using a vertical evaporation-based self-assembly (VESA) approach (see Figure 2a for a schematic of the setup). The assembly process involved immersing a substrate vertically or at an angle into a colloidal lignin suspension, where an evaporation-induced fluid flow moved the particles to the meniscus. As with other colloidal systems, achieving full large-area uniformity remained a challenge due to local variations in evaporation rate and flow dynamics.^59^ Nonetheless, the approach reliably produced regions with structural color and offered the potential for further optimization.

**Figure 2:**
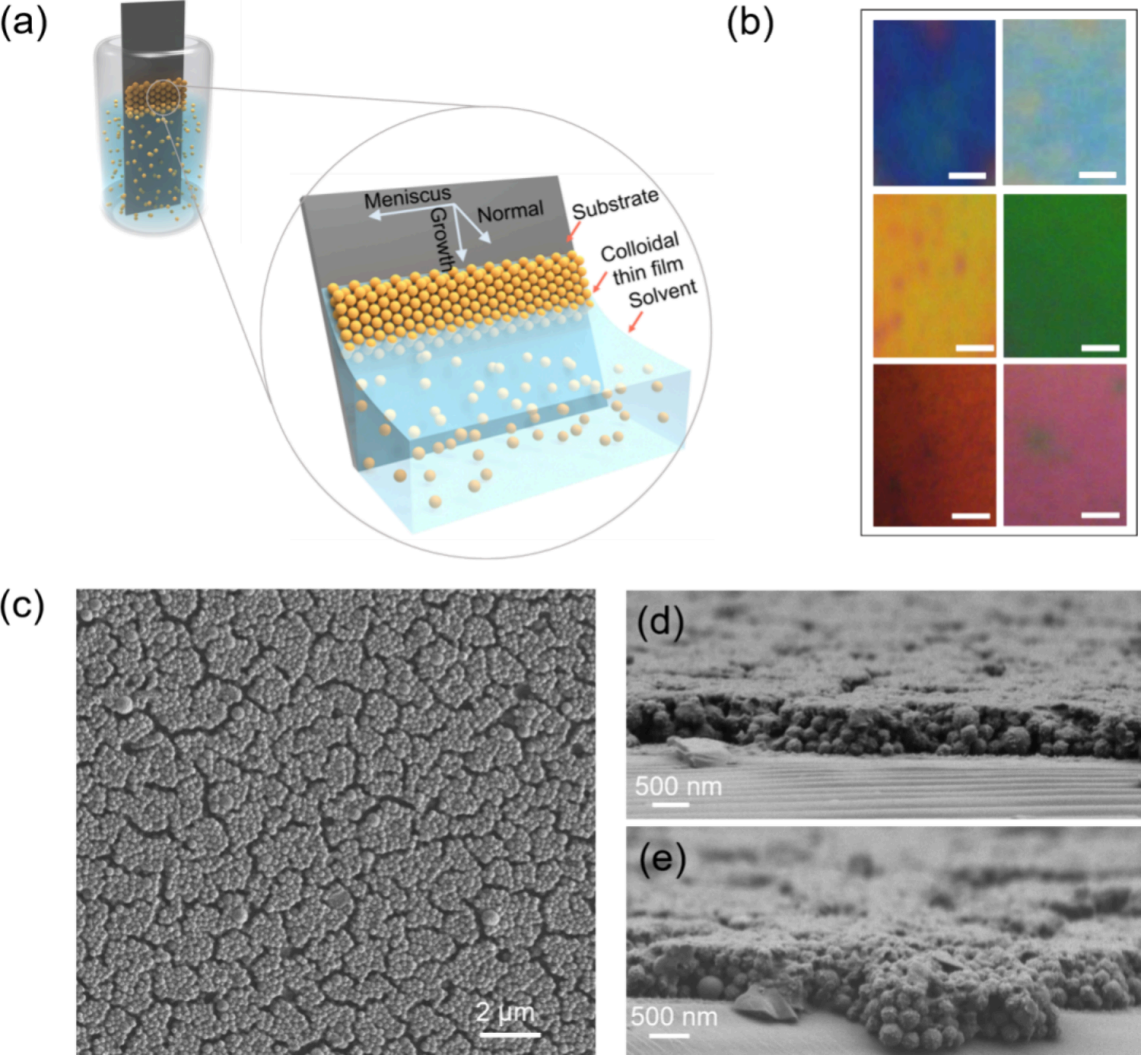
(a) Schematic illustration of VESA of LNPs thin film. (b) Interference optical micrograph showing interference colors by reflective LNP samples caused by different layer thicknesses. The length of the scale bars is 50 μm. (c) Top-view SEM images of the structure of LNPs films. (d,e) SEM cross-sectional images of the green and orange/red film, respectively.

The concentration of the LNPs solution was crucial for controlling the uniformity of the films. Although achieving uniform color across the entire 1 × 1 cm^2^ area (Figure S4) was challenging due to the changing concentration during evaporation, the films were sufficiently uniform over smaller areas (approximately 1 mm) for effective optical and morphological characterization with concentrations of 1.0 mg/mL at an evaporation rate of 0.50–0.55 mm/h. The VESA method resulted in different regions with vivid structural saturated colors, for example, green, yellow, orange, and red shown in Figure 2b. These films exhibit long-term stability, and no changes were detected in their structural color over a period of 1 year under ambient conditions. Although prone to mechanical wear and surface scratches, their durability can be improved with biobased protective coatings for practical applications.^68^ The strong saturation of the colors could be related to lignin’s UV absorption (Figure 1d), which leads to a gradually decreasing broadband absorption also at longer wavelengths. This broadband absorption reduces incoherent scattering, which can otherwise result in pale colors. Systems using polymeric particles typically lack sufficient absorption to achieve high color saturation,^14,69^ often requiring the addition of broadband absorbers like carbon black to suppress incoherent scattering and enhance vibrancy.^70–72^ In contrast, lignin naturally achieves this effect as a single-component material, eliminating the need for supplementary absorbers and providing a streamlined approach to producing saturated structural colors. The observed variation in color across different areas could potentially arise from variations in the thickness between these regions. We first examined a top-view SEM image of the red region sample revealing closely packed structures with distributed cracks that appeared across the surface, likely due to shrinkage during the drying process (Figure 2c). Figure 2d,e, along with additional cross-sectional SEM images (Figure S5), shows regions with varying thicknesses observed across different areas of the sample. The measured thicknesses across these areas range approximately from 370 nm to around 1 μm, depending on the location. Such variability likely corresponds to domains with different numbers of nanoparticle layers, which may range from approximately 2 to 5 layers based on the average nanoparticle size of ∼240 nm. However, the random close-packed arrangement of particles, combined with challenges in achieving perfectly vertical cross sections during sample preparation and inherent variability in the fracture angles, introduces uncertainty into these measurements. While these thickness estimates are broadly consistent with the observed color differences in the green and orange regions, precise alignment between optically identified regions and SEM measurements remains challenging.

Microreflectance spectroscopy enabled us to collect more details of the optical response for regions of different colors.^22,73–75^ Microreflectance spectra were recorded using a custom-built setup following the schematic detailed in Figure 3a. The resulting experimental reflectance spectra from differently colored regions (Figure 3b and Figure S6) exhibited distinct peaks in the visible spectrum, with maxima at approximately 623, 531, and 474 nm, corresponding to vibrant structural colors of orange/red, green, and blue, respectively. To confirm that the observed structural colors were not limited to a single particle size, we also fabricated thin films with larger particles (400 nm), which also produced similar vibrant colors (Figure S7). The reflectance spectrum for the blank silicon wafer showed no peaks, demonstrating that the colors observed from the lignin colloidal films were not caused by the silicon substrate (Figure S8). As another set of control experiments, we deposited the same colloidal nanoparticles on a transparent glass substrate (Figure S9). The observed structural colors were very faint, with reflectance below 10%, visible only under a microscope (Figure 3c). These results indicate that the nanoparticle layers did not act as effective photonic crystals with strong reflective structural colors on their own but that reflection at the substrate was essential for the optical response. In turn, reflection at the interface between the nanoparticle layer and glass was low due to the low refractive index contrast between the effective refractive index of the nanoparticle film (∼1.37) and glass (∼1.52). The value ∼1.37 was estimated using effective medium approximation,^76^ which accounted for the contributions of both nanoparticles (*n* ≈ 1.55) and air inclusions in the thin-film structure. To further understand the origin of the observed colors and their variations across the samples, we applied the transfer matrix method (TMM)^77–79^ to model the optical response of lignin films with varying thicknesses (see details in Supporting Information §1). We used the complex refractive index of lignin from Hollertz et al.^80^ and accounted for air inclusion effects through effective medium theory.^76^ The simulations showed optical responses similar to the experimental results by adjusting only the film thickness as a fitting parameter (Figure 3d). The thickness values obtained from this fitting were 294 (blue line), 550 (green line), and 804 nm (red line), which matched with the thickness range measured by SEM imaging. Minor differences between the experimental and simulated spectra could be attributed to nanoparticle scattering and local variations in the packing density and thickness, none of which were explicitly included in our model. Subtle contributions from local structural differences such as layer-to-layer arrangement or local stacking variations may subtly influence the optical response in certain domains, as reported in ultrathin colloidal systems.^81,82^ We assumed a uniform filling fraction and a constant film thickness, neglecting particle–particle interactions and local density variations. These approximations were reasonable within the scope of this study. As noted by Stavenga, the spectral shape and peak positions may also be influenced by variations in film thickness and possible absorption effects.^83^ Converting the spectra to Commission Internationale de L’Eclairage (CIE) chromaticity coordinates showed nearly covering the entire visible spectrum, as shown in the CIE diagram (Figure 3e). Based on the structural analysis, reflection measurements, and theoretical modeling, we conclude that the coloration in the lignin colloidal films arose primarily from thin-film interference as depicted in Figure 3f, where an increase in thickness caused a shift in color from blue to red, highlighting how the thickness influenced the observed reflected color.^84^ With a limited thickness of only a few layers, as shown by cross-sectional SEM and TMM modeling, and a strong dependence on the substrate’s reflectivity, the film lacked the thickness required for an effective photonic crystal effect (Figure S10). While slight arrangement effects may contribute, thin-film interference remained the dominant mechanism, with the reflective silicon substrate enhancing color visibility.

**Figure 3:**
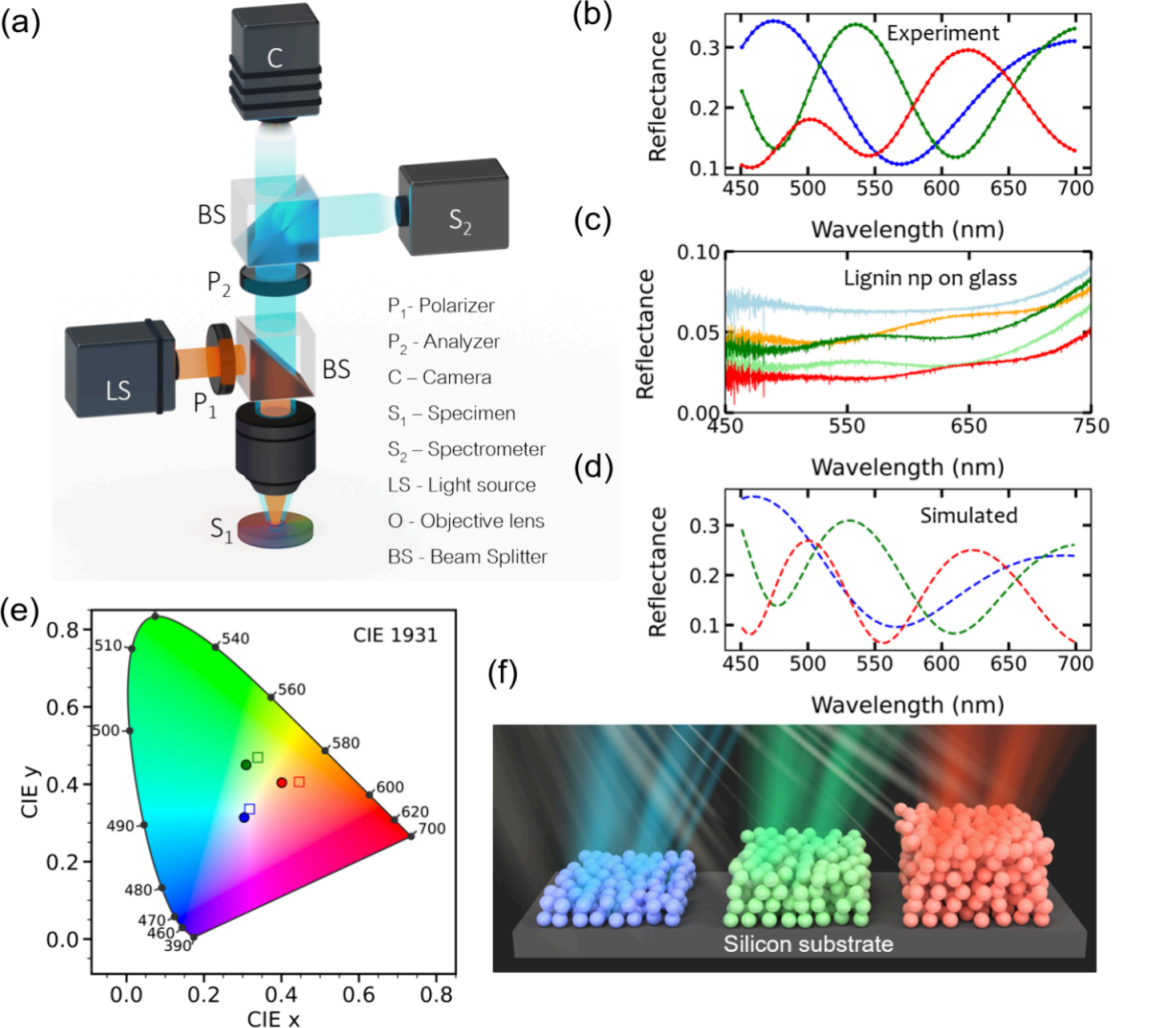
(a) Schematic of the microspectroscopy setup to measure microreflectance spectra. (b) Experimental reflectance spectra of different colored regions as a function of wavelength. (c) Reflectance spectra composed of 240 nm lignin particles prepared on a clear glass plate. Microreflectance spectra of different domains, showing broad optical peaks. (d) Simulated reflectance shows the effect of thickness on structural color where dashed lines represent the fit of the experimental data to a TMM model. (e) CIE chromaticity chart corresponding to the experimental data (hollow squares) and the simulated data (solid circles). (f) Proposed schematic of structural color production via light interference in multilayered LNPs, showing how the color varies with the thickness of the nanoparticle layers.

Of all wood polymers—cellulose, hemicellulose, and lignin—lignin has the lowest equilibrium moisture content at various relative humidities.^85^ This is related to the molecular structure of lignin, and the hygroscopic character can be further reduced by chemical modification of the hydrophilic functions groups (phenolic and aliphatic hydroxyl, methoxyl, carbonyl, and carboxyl groups) that are responsible for the hygroscopic nature of lignin. In this study, kraft lignin is acetylated to reduce the availability of these hydrophilic −OH groups (Figure 1c), making it soluble in chloroform. This chemical modification lowers water uptake and reduces material swelling with water vapor, leading to smaller modulation in the thickness during humidity exposure.^86,87^ However, water vapor does not only swell the matrix material, it is also adsorbed as a monolayer (Langmuir adsorption) in interior pores/air-pockets.^88^ Additionally, water fills the pores through capillary condensation at high RH. In Figure 4a and Figure S11, we plot the measured water content (as a percentage of the dry mass) against different RH values and fit the data with different sorption models: Henry’s law, Langmuir, and clustering, as well as a combination of these models.^89^ The curve shape partially resembles a type II isotherm and shows how the pore filling relates to spectral peak shift. The DVS curves initially rise sharply, corresponding to monolayer adsorption, and then increase linearly at intermediate RH. The subsequent rapid increase at very high RH reflects a capillary-driven pore filling behavior. The total water uptake at 100% RH in the lignin matrix material only, as calculated from Henry’s law, is 11.2% of the dry mass. However, water uptake in the films also depends on the packing density of the nanoparticles as voids between particles contribute to Langmuir sorption and capillary condensation effects. By reducing voids and forming a solid acetylated lignin thin film, these effects can be minimized, potentially lowering the total water uptake by more than 50% at high RH (Figure 4a). Another approach to reducing humidity sensitivity can be to coat the LNP films with a high-water barrier layer,^68^ which could further enhance the film’s stability in fluctuating environments.

**Figure 4:**
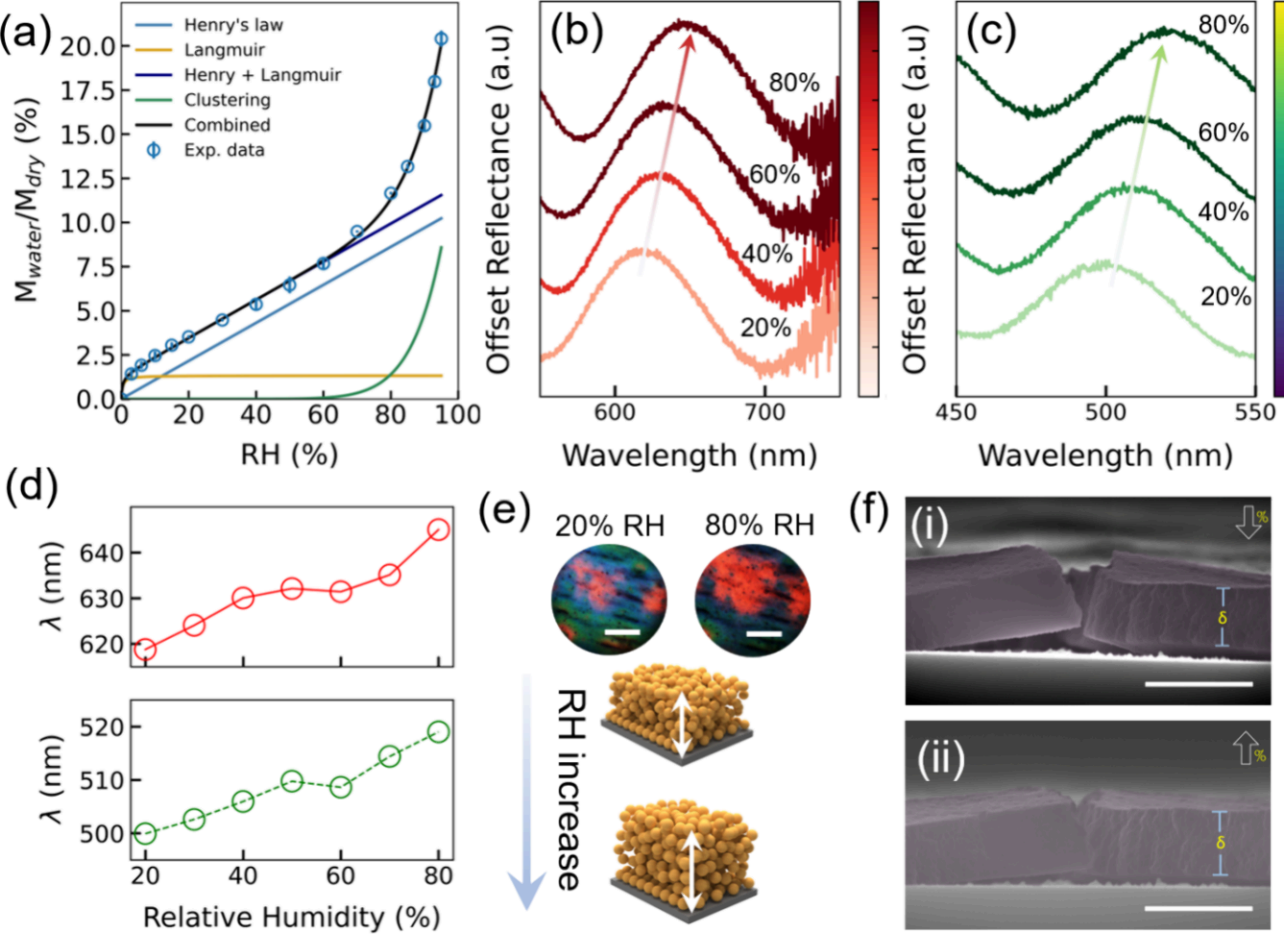
Colorimetric and structural responses of LNPs thin films under varying relative humidity (RH). (a) Water uptake during sorption, as a percentage of dry mass, is plotted against RH, fitted using different sorption models. (b,c) Reflectance spectra and optical images show dynamic color changes from greenish-pink at 20% RH to dark red at 80% RH, with two distinct peaks shifting due to different orders of interference. The reflectance values on the *y*-axis are offset vertically for clarity; actual reflectance values are represented in percentage. (d) The peak positions for both orders shift nonlinearly as RH increases, reflecting the swelling of the film. (e) Optical microscopy images and a schematic illustrate the structural changes in the film as it absorbs water and swells. Scale bars: 100 μm. (f) Environmental SEM (ESEM) cross-sectional images show swelling of LNPs at different water vapor pressures. The images are false colored purple (lignin film) for clarity. Scale bars: 5 μm.

Using a sorption value of 11.2%, the maximum volume change of the polymer film can be calculated using the following equation:

$$\frac{V_{w}}{V_{0}}=\left(\frac{m_{w}}{m_{0}}\right).\frac{\rho_{o}}{\rho_{w}}$$ 1

where the ratio of water absorbed by the lignin matrix to the dry mass (*m*_*w*_/*m*_0_) is 11.2% at 100% RH, and the densities of the lignin (ρ_*o*_) and water (ρ_*w*_) are 1350 and 998 kg/m^3^, respectively. This leads to a total moderate volume increase of 15.2% for the matrix material, which is an increase in lignin particle diameter of 4.8%, indicating limited swelling expected for acetylated lignin.

To investigate how the structural color of the lignin colloidal thin film responds to varying relative humidity levels, we exposed the film to different humidity conditions and analyzed the corresponding spectral shifts. The area with a pinkish-green color domain was selected for the humidity response study due to its pronounced color variability and ability to observe peak shifts within the visible spectrum. Microreflectance spectra (Figure 4b,c) revealed two distinct optical resonances (interference peaks) that shift toward longer wavelengths as humidity increases. Specifically, the lower-order peak shifts from 618 nm at 20% RH to 645 nm at 80% RH, while the higher-order peak moves from 499 to 519 nm (Figure 4d). This corresponds to wavelength shifts (Δλ) of 27 and 20 nm, respectively, indicating modest optical changes as a result of the film’s limited swelling behavior and limited water uptake (condensation) in pores within the tested relative humidity range. Indeed, although we note that the peak position shift increases more rapidly at relative humidities above 60% (up to 80%RH) due to water clustering in pores, the shift is still modest. The behavior in peak shifts at intermediate to high relative humidities suggests nonlinear increases in optical thickness (τ = *n*_eff_. *d*), caused by the mechanism of water uptake (see the DVS results). Along with the spectral shift, the small variation in percentage reflectance suggests that the material’s response to humidity is likely due to both limited water uptake and small variations in the refractive index difference (Δ*n*) (see Figure S12).^78^ These combined effects help maintain the stability of the optical properties across 20–80% RH range. Figure 4e presents a schematic illustration and optical microscopy images of the polymer film under two different humidity conditions. The optical images demonstrate a color change from greenish-pink at 20% RH to dark red at 80% RH, highlighting the film’s humidity-responsive nature.

We further utilized ESEM to directly observe the swelling of the thicker lignin thin-film sample due to variations in humidity, as shown in Figure 4f. The RH was controlled using water vapor pressures of 857 Pa (79% RH) and 245 Pa (20% RH), both at a temperature of 10 °C. Side-view ESEM images revealed swelling in the order of ∼5% in thickness. Note that this is an approximation in thickness increase, due to difficulties in exactly imaging and measuring the thickness of the cross-section. For the same reason, a thicker film (diameter of LNP approximately 400 nm) was chosen for the swelling investigation by ESEM. This differs from the reflection measurement presented earlier in Figures 4b,c, and 3, which were based on the thin colloidal film (nanoparticle diameter ∼240 nm). The swelling led to the disappearance of cracks in the film and, in some areas, delamination from the substrate. The process was found to be reversible as the film returned to its original state with a decrease in humidity. The LNPs film showed color changes with varying humidity, without requiring additional hygroscopic materials or fillers, and we can quantify the sensitivity of the color shift to water uptake as follows:

S=Peakshift(nm)/Wateruptake(%) 2

For the lignin films, 11.2 wt % water sorption resulted in a 26.2 nm peak shift for the lower wavelength peak (near 500 nm), yielding a sensitivity of 2.3 nm/wt%. The higher wavelength peak (close to 600 nm) showed a 28.6 nm peak shift, giving a sensitivity of 2.5 nm/wt%. These results show noticeable optical changes in response to humidity within the 20–80% RH range. The reversibility of the structural color response upon cyclic humidity changes is included in Figure S13 in Supporting Information, which shows that the peak position shift of a colored lignin film is reversible with minimal hysteresis, suggesting that water sorption occurs without leading to irreversible material degradation within the RH interval 20–80%.

## Conclusions

In summary, we have demonstrated the structural colors of colloidal lignin-based thin films produced via evaporation-induced self-assembly and show that their colors vary with RH. By controlling the film thickness, this study shows that lignin can yield a range of colors without requiring supplementary absorbers, presenting an ecofriendly and biocompatible alternative to traditional synthetic photonic pigments. Our results show that the lignin thin films exhibit modest but measurable color shifts in response to humidity changes. Future work may focus on refining the self-assembly processes for lignin to achieve large-scale, uniform films, extending its application to multifunctional photonic ink and sustainable products. Further studies could also examine alternative assembly techniques and explore modifications in LNP morphology to expand the color range and functionality.

## Materials and Methods

### Synthesis of Colloidal Lignin

The softwood Kraft Lignin (KL) was obtained from the Lignoboost process^90^ and originated from Stora Enso (Finland). The KL was washed (acidic water, pH 2) and sequentially solvent-fractionated using EtOAc, EtOH, MeOH, and acetone following a previously developed protocol.^42,43^ Only the derived acetone lignin fraction was used in this study, and it had a molecular weight of ∼5400 g mol^–1^ and PDI of 2.^91^ The FT-IR spectrum of the acetone lignin raw materials is included in Figure S2 in the SI file. The acetylation of lignin was conducted as described in earlier works.^49,50^ SDS and chloroform were purchased from Sigma-Aldrich, and isopropanol was purchased from VWR chemicals and used without further purification. Solid lignin particles were prepared by dissolving 80 mg of acetylated lignin in 2.2 mL of chloroform with overnight stirring. All of the hydrophobic phase was then passed through a hydrophilic ceramic membrane with a pore size of 200 nm into a continuous water phase of 20 mL of 3.9 mM SDS solution in Milli-Q water, using a transmembrane pressure of 30 kPa. The setup involved a cylindrical SPG (Shirasu porous glass) membrane (10 mm wide, 20 mm long, with 200 nm pores) enclosed in a pressure-sealed stainless-steel module (Figure S1 in SI). This formed an O/W emulsion. The resulting suspension of oil droplets was then stirred at 500 rpm overnight to evaporate the chloroform, forming solid lignin particles. The excess surfactant was removed using dialysis (cellulose dialysis membrane, molecular weight cutoff of 14 kDa) against Milli-Q water for 3 days. After dialysis, the particles were dried in an oven at 40 °C. Subsequently, they were redispersed in a Milli-Q water for further experiments.

### VESA of Lignin Colloids on Silicon Wafer

Silicon wafers (1 × 1 cm^2^) were cleaned using a multistep process. Initially, the wafers were immersed in a 0.5 wt % NaOH solution and ultrasonicated for 5 min. They were then washed with deionized water for another 15 min, followed by a 15 min rinse with acetone. After drying, the silicon substrates underwent a piranha treatment, consisting of a mixture of 3 parts sulfuric acid (H_2_SO_4_) and 1 part hydrogen peroxide (H_2_O_2_). Lignin nanoparticles were prepared by ultrasonicating a 1 mL aqueous suspension of the particles at a concentration of 1.0 mg mL^–1^ for 15 min. The prepared suspension was transferred to a plastic cuvette, and a clean rectangular silicon wafer piece was placed vertically in the solution. The assembly process was conducted at 55 °C in the oven until all of the water evaporated. As a control, the self-assembly of LNPs was also performed on piranha-cleaned transparent glass substrates.

### Characterization

#### SEM

SEM micrographs were captured using a Hitachi SEM S-4800 (Japan) at an accelerating voltage of 1 kV. Samples were prepared by drop-casting a 5 μL suspension onto a cleaned silicon substrate. Initially, 1 × 1 cm^2^ silicon wafers were thoroughly cleaned with a piranha solution. After being cleaned, the particles were deposited onto the surface, and the sample was left to dry overnight at room temperature.

#### TEM

TEM images were taken with a JEOL JEM-2100f TEM (JEOL, Japan) operating at 200 kV. To prepare the TEM samples, a concentration of 0.01 mg/mL of lignin nanoparticles in Milli-Q water was used, and 1–3 μL of this solution was deposited onto carbon-coated copper grids and allowed to dry.

#### FT-IR

FT-IR spectra were collected using a PerkinElmer Spectrum 2000 FT-IR equipped with a MKII Golden Gate single-reflection ATR system, recording spectra in the range of 600 to 4000 cm^–1^ with an average of 32 scans at a resolution of 4.0 cm^–1^ at room temperature.

#### DLS & UV-Vis

DLS was performed using a Zetasizer Nano ZS instrument (Malvern Panalytical) to determine the hydrodynamic diameter distribution of LNPs in Milli-Q water.

UV–vis spectra were collected using a UV–vis spectrophotometer (Shimadzu UV 2550) in the wavelength range of 300–800 nm. Measurements were performed at 40% RH and 21 °C.

#### ESEM

ESEM was obtained using a FEI Thermo Scientific Quattro. The cross-sectional imaging was conducted without sputter coating, under varying humidity conditions. A thicker lignin film sample was selected for these measurements to enable a clearer observation of thickness changes during swelling. Initially, the ESEM chamber was evacuated to approximately 10^–5^ Torr before introducing water vapor. Once the desired pressure was achieved, the system was stabilized for 5 min prior to observation. Different water vapor pressures ranging from 1 to 10 Torr, which correspond to RHs of 10 to 80% at 24 °C, were tested. Additionally, dry nitrogen was introduced at pressures between 1 and 10 Torr for a comparative analysis. However, due to poor imaging quality, nitrogen pressures above 10 Torr were not used. Thickness changes were measured by averaging measurements from multiple locations on the sample.

#### Dynamic Vapor Sorption Measurements

Water sorption and desorption measurements were performed using a DVS Advantage (Surface Measurements Systems Inc.) at 21 °C (0–95% RH). Equilibrium was achieved when dm/dt <0.002% for >5 min or a maximum of 360 min per step.

#### Optical Measurement

Optical microscopy images and reflectance spectra were obtained using a custom-made microspectroscopic setup combined with a custom-built humidity chamber. The samples were studied under controlled RH, and desired RH was achieved by connecting pressurized nitrogen cylinder together with a P-series humidifier from Cellkraft AB, Stockholm, Sweden. A 50W LED/halogen lamp illuminated the sample area, which was viewed through a Motic BA310 Pol microscope with a Moticam ProS5 Lite camera. The reflected light was collected from the camera port via an optical fiber connected to a Thor Laboratories CCS 200 M spectrometer. An objective lens with ×40 magnification (NA = 0.65) was used to record the spectra. All reflectance data were normalized to a Thor Laboratories silver mirror (ME1-P01), and dark (0% reflectance) measurements were collected by shunting the light to the spectrometer port.

Additional optical microscopy images and reflectance spectra at ambient room conditions were obtained using another custom-made microspectroscopic setup, similar in design to the one used for humidity-controlled measurements. Here, the samples were illuminated using a 50 W halogen lamp attached to a Nikon Eclipse L200N optical microscope (Japan). The reflected light was collected from the camera port with an optical fiber connected to an Ocean Optic HR 4000 spectrometer (USA). An objective lens with a ×50 magnification (NA = 0.80) was used to record spectra.

## Supplementary Material



## Notes

Information of TMM for optical simulations; photograph of the SPG membrane setup; FT-IR spectrum of the lignin acetone-fraction raw material; size distribution using DLS; digital photograph of LNP deposited thin film on silicon wafer; cross-sectional SEM image; microreflectance spectra of lignin colloidal thin films; FE-SEM micrograph LNPs and microreflectance of lignin thin films assembled from 400 nm-diameter LNPs; microreflectance of silicon wafer; lignin colloidal thin films on glass substrate; colormap showing the dominant optical mechanism; dynamic vapor sorption; reflectance maxima at various humidity levels; reversible structural color response to humidity cycling (PDF)
