# Supplementary material for: Structurally Colored Thin Films Based on Acetylated Lignin Nanoparticles
Source: ACS Nano. 2025 Jul 1;19(27):24713–23. doi: 10.1021/acsnano.4c16679 (PMC12269353; doi:10.1021/acsnano.4c16679)
Supplement: Supplementary file 1 [file nn4c16679_si_001.pdf]

Supporting information for publication

Structurally Colored Thin Films based on Acetylated Lignin Nanoparticles

Ravi Shanker[1*], Anran Mao[1], Longzhu Liu[2], Aseem Salhotra[1], Yuxiao Cui[1], Bang An[1], Magnus Jonsson[2], Anna J. Svagan[1]*
E-mail: shanker@kth.se, svagan@kth.se

[1]Royal Institute of Technology (KTH), Dept. of Fibre and Polymer Technology, SE-100 44 Stockholm, Sweden

[2]Laboratory of Organic Electronics, Department of Science and Technology, Linköping University, SE-601 74 Norrköping, Sweden

Keywords: Structural colors, lignin, nanoparticles, thin film interference, membrane emulsifications, sorption

This file includes:

Pages 1-11

Section §1

Figures S1-S13

§1. Transfer matrix method for optical simulations

We apply the transfer-matrix method, following Byrnes et al., to model light reflection/transmission through the multilayer system composed of lignin with air and Si as surrounding media (see Schematic§1). The lignin nanoparticle film is modeled as a composite material, where the effective refractive index n_{eff} is calculated using Maxwell-Garnet effective medium approach. This theory considers the refractive index of the nanoparticles ($n_{eff} \approx 1.55$) and air ($n_{air} \approx 1.0$), as well as their respective volume fractions $\left(f_{lignin}\ and\ f_{air} \approx 1 - f_{lignin}\right)$. The effective permittivity ε_{eff} is expressed as:

$$\varepsilon_{eff} = \varepsilon_{air} \cdot \frac{1 + 2c_F}{1 - c_F},$$

Where $c_F = f_{lignin} \cdot \frac{\varepsilon_{lignin} - \varepsilon_{air}}{\varepsilon_{lignin} + 2\varepsilon_{air}}$. Here f_{lignin} is the volume fraction, ε_{lignin} is the permittivity of lignin, and ε_{air} is the permittivity of the air. The effective refractive index (n_{eff}) is then derived as:

$$n_{eff} = \sqrt{\varepsilon_{eff}}$$

Here, lignin nanoparticle volume fraction of f_{lignin}= 0.50 was assumed, consistent with reported values in similar nanoparticle system in the literature.[1,2] This assumption provides a realistic representation of the porosity in lignin-based nanoparticle assemblies and ensures reasonable agreement between the modeled and experimental reflectance spectra. The n_{eff} is incorporated into the transfer matrix method to represent the refractive index of lignin nanoparticle layer defined as n_i.

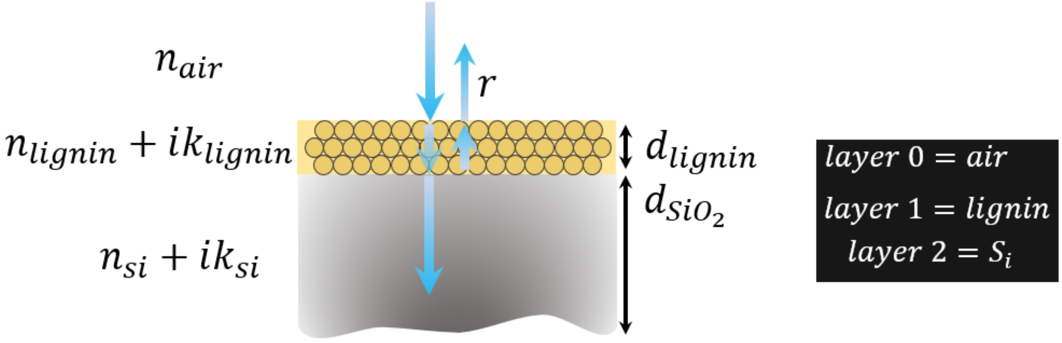

Schematic §1. Light propagation through a multilayer system, including air, lignin, and a silicon substrate, with r and t representing reflection and transmission coefficients for the total system.

The multilayer system now consists of air, the lignin film, and a silicon substrate. The reflection (r) and transmission (t) coefficients are derived by considering the behavior of light at each interface (air-lignin and lignin-silicon) and its propagation through the lignin. The relationship between the incident, reflected, and transmitted light amplitudes is governed by the overall matrix M, which represents the system's optical behavior:

$$\begin{pmatrix} 1 \\ r \end{pmatrix} = M \begin{pmatrix} t \\ 0 \end{pmatrix}$$

where M is a 2 x 2 matrix that contains all the information about the light reflection and transmission for the entire multilayer system. The matrix M is constructed by multiplying the matrices that account for light reflection and transmission at each interface, as well as the propagation of light through the polymer layer. The overall transfer matrix M is:

$$M = T_{01} D_1 T_{12}$$

Where T_{01} represents the reflection and transmission at the air-lignin interface. D_1 represents the phase change due to propagation through the lignin layer. T_{12} represents the reflection and transmission at the lignin-silicon interface. At each interface between layers i and $i + 1$, under normal incidence, a transfer matrix $T_{i,i+1}$ can be defined as follows:

$$T_{i+1} = \begin{pmatrix} 1/t_{i,i+1} & r_{i.i+1/}/t_{i,i+1} \\ r_{i,i+1}/t_{i,i+1} & 1/t_{i,i+1} \end{pmatrix}$$

Where the reflection $(r_{i,i+1})$ and transmission $(t_{i,i+1})$ coefficients are given by

$$r_{i,i+1} = \frac{n_i - n_{i+1}}{n_i + n_{i+1}};$$

$$t_{i,i+1} = \frac{2n_i}{n_i + n_{i+1}};$$

where n_i and n_{i+1} being the complex refractive index layers i and layer $i+1$, respectively.

$$D_i = \begin{pmatrix} e^{-i\delta_i} & 0 \\ 0 & e^{i\delta_i} \end{pmatrix}$$

Where phase change δ_i of light propagating in a layer of thickness d_i is expressed as $\delta_i = 2\pi n_i d_i/\lambda$, with λ being the wavelength of light. Once the overall transfer matrix M is calculated, the reflection coefficient r for the system can be determined using:

$$r = {}^{M_{10}}/_{M_{00}}$$

whereM_{00} and M_{10} are the elements of the overall matrix M, which takes the form:

$$M = \begin{pmatrix} M_{00} & M_{01} \\ M_{10} & M_{11} \end{pmatrix}$$

The reflectance R, which represents the fraction of light reflected by the multilayer system, is given by: $R = |r|^2$.

Shirasu Porous Glass (SPG) membrane setup

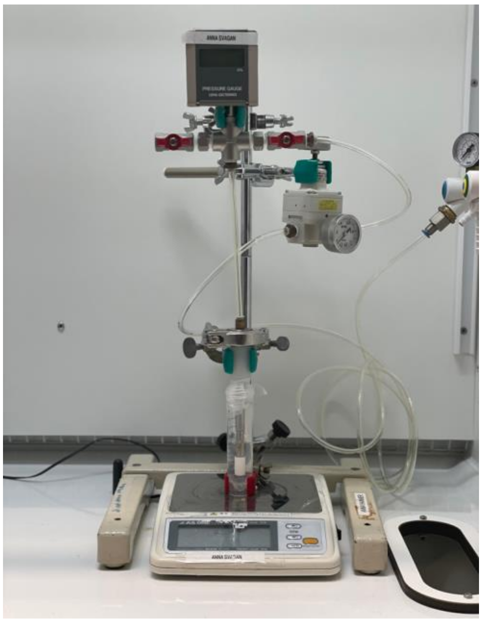

Figure S1. Digital photograph of the Shirasu Porous Glass (SPG) membrane emulsification device.

FTIR spectrum of the lignin acetone fraction raw material

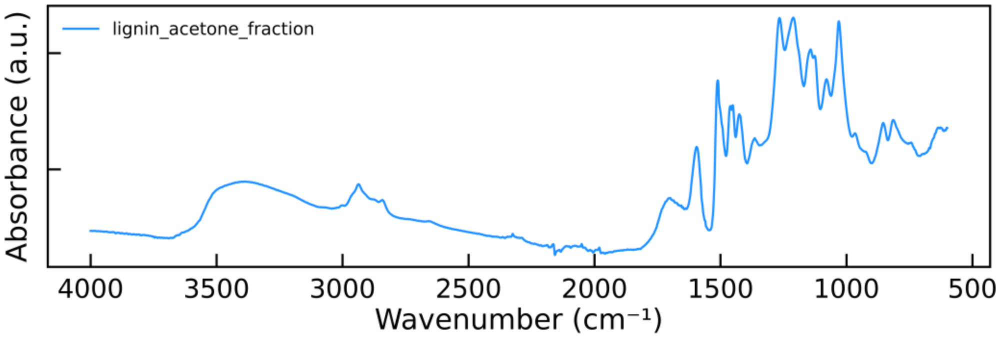

Figure S2. FTIR spectrum of the raw lignin acetone fraction.

Dynamic light scattering

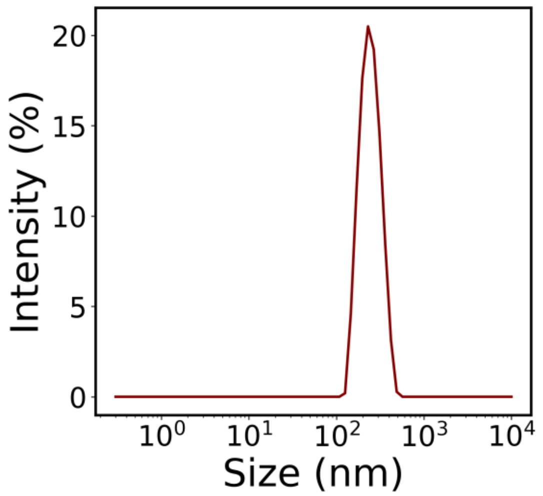

Figure S3. The size distribution (diameter) of lignin nanoparticles in deionized water (polydispersity index of 0.07), measured with dynamic light scattering.

Lignin thin film on Silicon wafer.

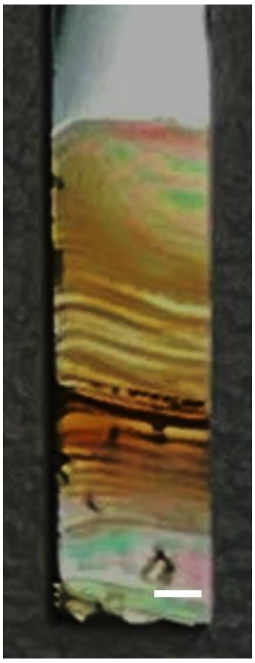

Figure S4. Digital photograph of lignin nanoparticle deposited thin film on silicon wafer. Scale bar: 1 cm.

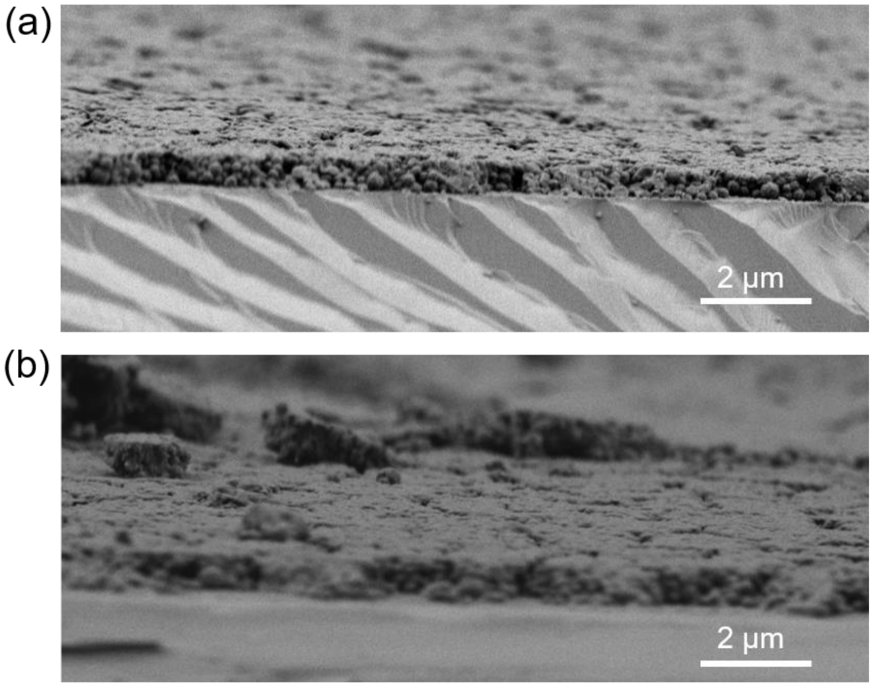

Figure S5. Cross-sectional SEM image showing a domain with peeling from the surface, resulting in localized regions with thickness variations. This highlights the non-uniformity in nanoparticle layer stacking across the sample.

Structural Color Palettes and micro reflectivity in lignin thin film

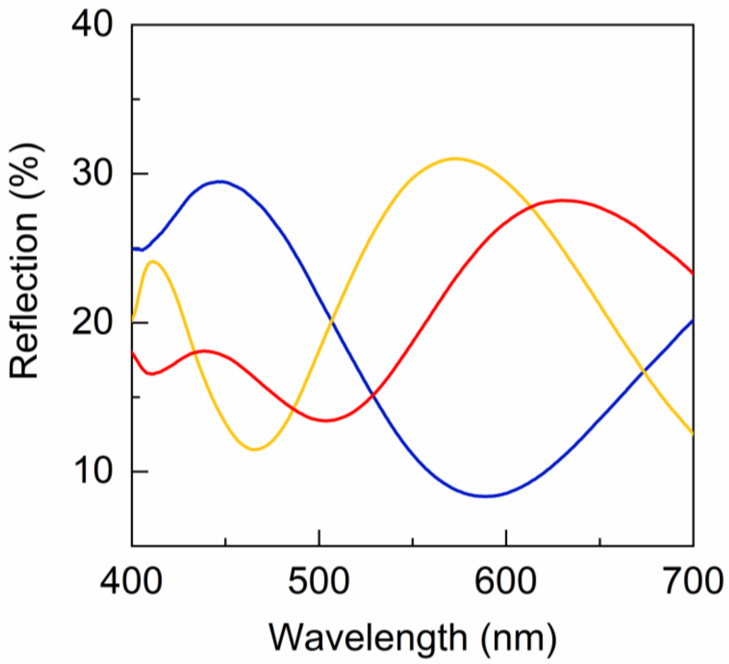

Figure S6. Micro-reflectance spectra of lignin colloidal thin films with different colors, corresponding to variations in film thickness.

FE-SEM Micrographs and Reflectance Spectra of LNPs Film (diameter ~ 400 nm)

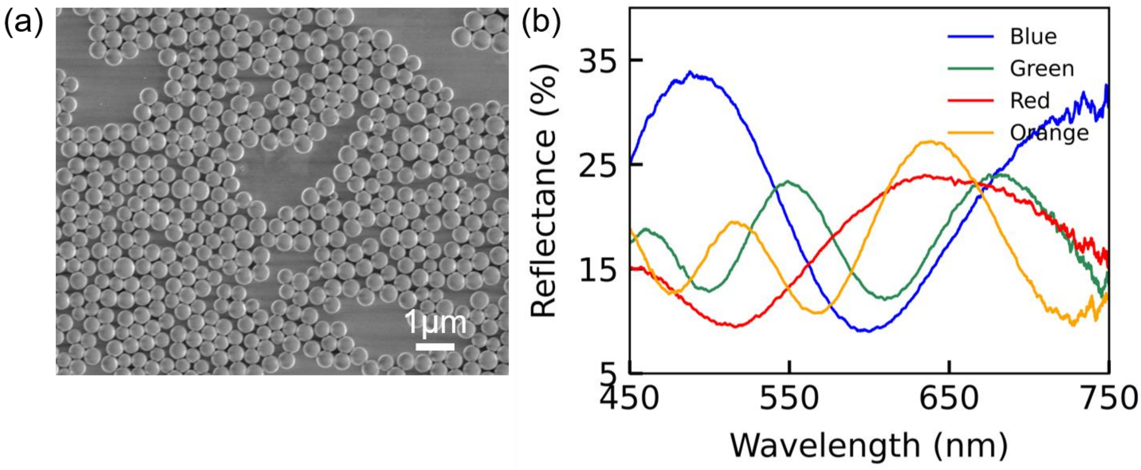

Figure S7. (a) FE-SEM micrographs showing LNPs. (b) Measured reflectance spectra across different colored regions of a film assembled from 400 nm diameter lignin nanoparticles.

Microreflectance of Silicon wafer

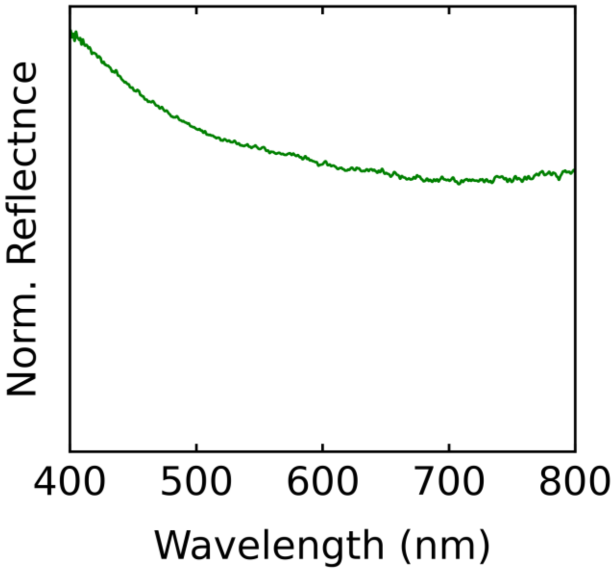

Figure S8. Specular reflectance of the silicon wafer measured using the microspectrophotometer.

Lignin colloidal thin films on glass substrate

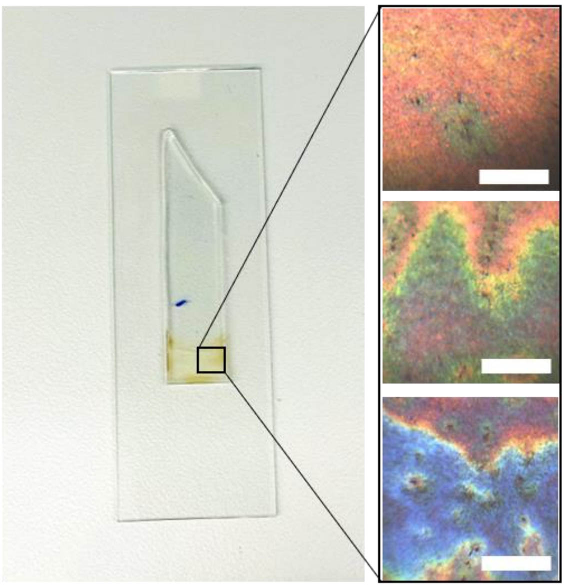

Figure S9. Optical microscopy images of a colloidal lignin thin film deposited on a transparent substrate. The zoomed-in images on the right highlight different structural color domains, which arise due to varying thicknesses across the film. The length of the scale bars is 100 microns.

Dominant Optical mechanism

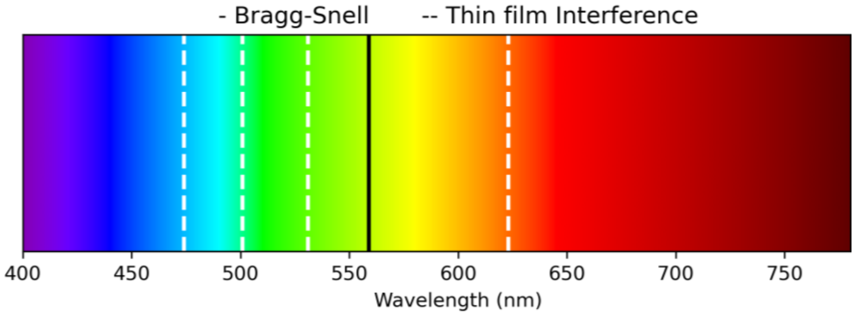

Figure S10. Comparison study to determine the dominant optical mechanism. The observed reflection peaks (dashed lines) are compared with the expected Bragg-Snell reflection (solid black line). The Bragg-Snell law predicts a primary reflection peak near 559 nm, which corresponds to the red-colored domain where measurements were taken. However, the sample also displays variations in thickness, resulting in green and blue color domains with peak positions at 531 nm and 474 nm, respectively. These additional resonances, including a main peak at 623 nm and a secondary peak at 501 nm, suggest that thin film interference is the dominant mechanism, as these values do not align with Bragg-Snell diffraction. The multiple resonances indicate behavior typical of thin film systems rather than a 3D photonic crystal.

Dynamic vapor sorption.

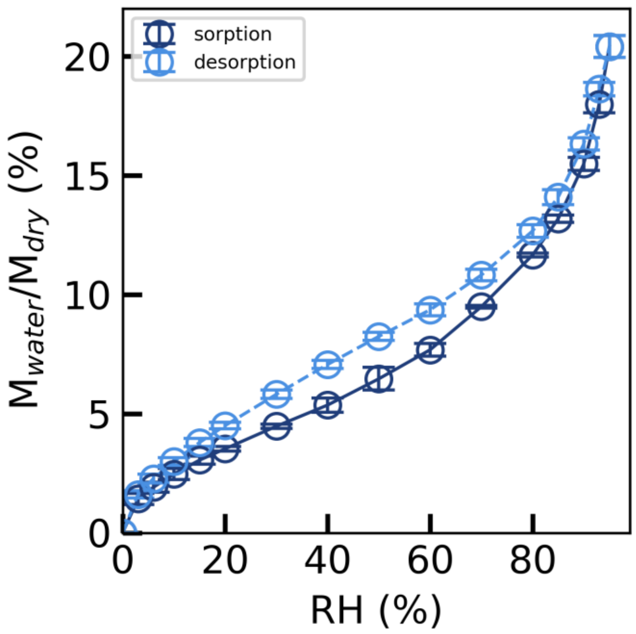

Figure S11. Water vapor sorption and desorption isotherms for lignin colloidal pellets at 21°C.

Humidity dependent reflection maxima behavior

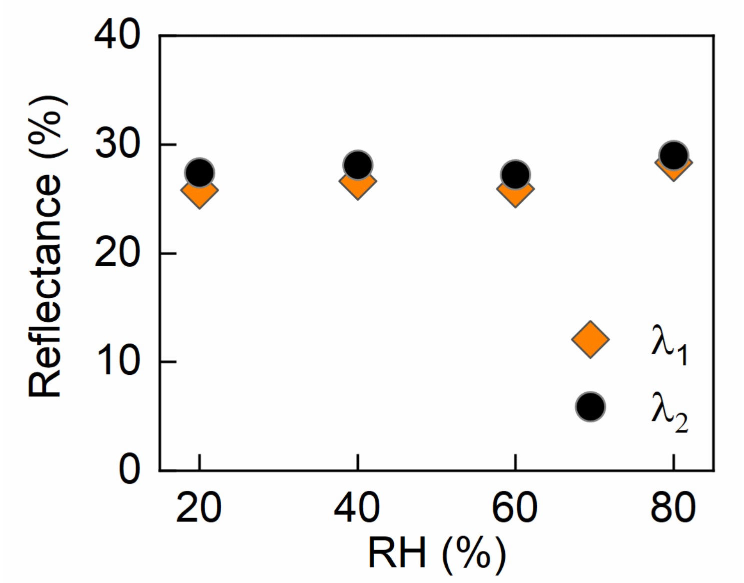

Figure S12. Reflectance maxima at various humidity levels, showing relatively constant reflection maxima with minor variations in percentage reflectance across the range.

Reversible structural color response to humidity cycling

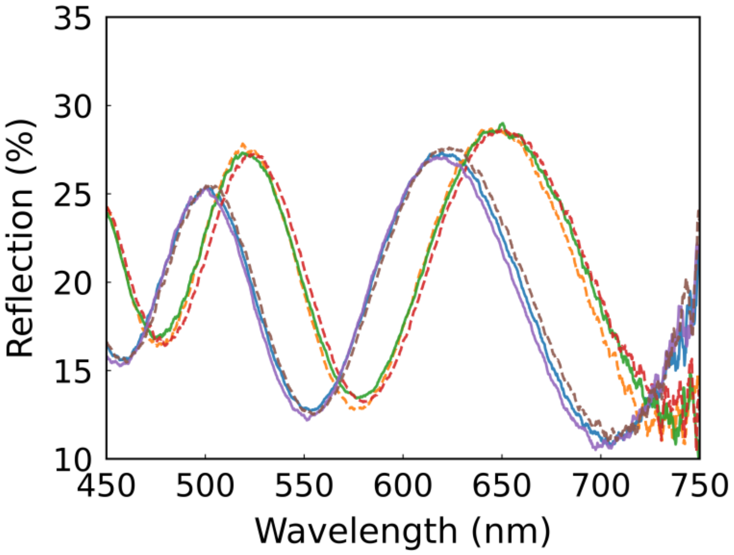

Figure S13. Reversibility of the structural color response upon cyclic humidity changes. Reflectance spectra were recorded while cycling the relative humidity (RH) between 20% and 80%.

The reversibility of the structural color response upon cyclic humidity changes is show in Figure S13. It reports the reflected spectra while cycling the RH between 20% to 80% for three consecutive cycles. The peak position shifts are minimal, and we note that the acetylation of lignin, leads to minimal swelling of the nanoparticles; with an expected lignin particle diameter increase of 4.8% at 100%RH indicating limited nanoparticle swelling. Additionally, the pore sizes distribution, found in between nanoparticles, influences the water up-take, and for our nanoparticle structure we observed Langmuir sorption and, to a lesser degree, capillary condensation in the 20-80% RH range, see Figure 4a in the main manuscript.

References

1. Song, C.; Wang, P.; Makse, H. A. A phase diagram for jammed matter. *Nature* **2008**, *453*, 629–632.
2. Xiao, M.; Li, Y.; Allen, M. C.; Deheyn, D. D.; Yue, X.; Zhao, J.; Gianneschi, N. C.; Shawkey, M. D.; Dhinojwala, A. Bio-inspired structural colors produced via self-assembly of synthetic melanin nanoparticles. *ACS Nano* **2015**, *9* (5), 5454–5460
